# Toward Quantum-Informed Atom Pairs

**DOI:** 10.1021/acsomega.3c09744

**Published:** 2024-01-26

**Authors:** Bartłomiej Fliszkiewicz, Marcin Sajdak

**Affiliations:** † Faculty of New Technologies and Chemistry, Military University of Technology, Warsaw 00-908, Poland; ‡ Faculty of Energy and Environmental Engineering Silesian University of Technology, Gliwice 44-100, Poland; § School of Chemical Engineering, University of Birmingham, Edgbaston, Birmingham B15 2TT, United Kingdom

## Abstract

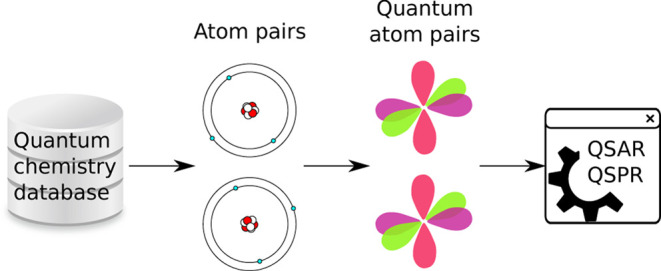

In the following research, a new modification of traditional atom pairs is studied. The atom pairs are enriched with values originating from quantum chemistry calculations. A random forest machine learning algorithm is applied to model 10 different properties and biological activities based on different molecular representations, and it is evaluated via repeated cross-validation. The predictive power of modified atom pairs, quantum atom pairs, are compared to the predictive powers of traditional molecular representations known and widely applied in cheminformatics. The root mean squared error (RMSE), *R*^2^, area under the receiver operating characteristic curve (AUC) and balanced accuracy were used to evaluate the predictive power of the applied molecular representations. Research has shown that while performing regression tasks, quantum atom pairs provide better fits to the data than do their precursors.

## Introduction

The use of quantitative structure–activity-property relationship (QSAR/QSPR) methods depends on the representation of molecular structures as numbers. Molecular descriptors are applied to transform chemicals to numerical representations. There are many different molecular descriptors, some of which encode properties of the compound, others try to capture the complexity of the molecule, etc. There are also molecular descriptors that rely on fragments of molecules, e.g., extended connectivity fingerprints (ECFPs),^1^ atom pairs,^2^ and topological torsion descriptors.^3^ In many cases, these types of descriptors transform molecules into vectors of the same length, either zeros or ones, called bits or vectors of the counts of each molecular fragment.

The application of molecular descriptors is limited not only to QSAR/QSPR but also to determining the similarity of molecules.^4−6^ The descriptors of continuous values may be put into a vector, and the similarity between two molecules can be computed using Euclidean distance. The bit representation vectors, such as the Tanimoto index, are the basis for calculating the similarity coefficients.

Since the introduction by Carhart et al., the idea of atom pairs has been modified several times^7−13^ and has been applied to construct various models. In this research, novel molecular descriptors, yet another modification of atom pairs, are proposed and examined for use in QSAR and QSPR applications. Traditional atom pairs are modified by associating various atom pairs with values taken from quantum-chemical calculations. To some extent, the method presented in this article is similar to the bond descriptors applied to bond dissociation energy introduced by Qu et al.^14^ that was applied by Raza et al.^15^ to model the C–F bond dissociation energy of PFASs.

## Materials and Methods

All procedures and analyses were conducted by using the Jupyter Lab^16^ environment, the Python^17^ programming language, and the following modules: RDKit,^18^ Scikit-learn,^19^ NumPy,^20^ Pandas,^21,22^ Matplotlib,^23^ and seaborn.^24^

### Quantum-Informed Atom Pairs

The RDKit implementation of atom pairs is based on their original definition by Carhart et al.^2^ The pair of atoms is described by the topological distance between the atoms in the pair; each atom is described by its element, the number of non-hydrogen atoms it is bonded with, and its number of π-bonding electrons. To enrich atom pairs with quantum-chemical properties, 1089 unique atom pairs with distances of up to 4 bonds were generated from the QM9-extended-plus^25^ database. Based on the set of unique atom pairs, two different approaches to define quantum atom pairs (QAPs) were utilized.

In the first definition, every atom pair was assigned the arithmetic means of the quantum properties of all of the molecules containing the atom pair. In the case of multiple occurrences of an atom pair in the molecule, the property value was divided by the number of occurrences. Since there are 11 quantum properties in the quantum property database, every atom pair was associated with 11 values. The above-mentioned quantum properties include the dipole moment, isotropic polarizability, energy of the highest occupied molecular orbital (HOMO), energy of the lowest unoccupied molecular orbital (LUMO), LUMO and HOMO energy differences, zero-point vibrational energy, internal energy at 0 K, internal energy at 298.15 K, enthalpy at 298.15 K, free energy at 298.15 K, and heat capacity at 298.15 K.

The alternative definition was to generate 10 binned histograms from the values of the quantum properties of molecules containing specific atom pairs divided by the number of occurrences of the atom pair. Thus, 110 different values were assigned to the atom pairs.

The modified atom pairs are intrinsically highly dependent on the composition of the quantum property database, which impacts the applicability domain of QAP. Due to the narrow group of elements present in the QM9-extended-plus database, the atom pairs are limited to C, O, N, F, S, Cl, and Br. This limitation is transferred to the applicability domain. In this study, molecules containing only atom pairs from the set generated from the QM9-extended-plus database were curated from experimental databases.

From the aforementioned QAPs, three sets of molecular descriptors were generated:

1. a sum of the quantum properties of the atom pairs present in the molecule −11 descriptors (sumQAP),
2. a sum of the histograms of the atom pairs −110 descriptors per molecule (hQAP),
3. a vector of quantum properties of atom pairs −11,979 values (spQAP).

### Experimental Databases

Modeling was conducted with 10 different experimental databases: 6 used for regression and 4 for classification tasks. Before any modeling, the data sets were truncated to include only those molecules belonging to the applicability domain. Table 1 summarizes descriptions of the databases and gives their references.

**Table 1:** Regression and Classification Task Data Sets Used in the Research^a^

database	*n*	min	max	units	reference
log *P*	5574 (8199)	–4.64	8.27		(26)
BACE-1 pIC50	285 (1513)	2.699	10.523	μM (log)	(27,28)
solubility	803 (1128)	–11.6	1.58	mols per liter (log)	(28)
lipophilicity	2237 (4200)	–1.50	4.48		(28)
ionization energy	1575 (2147)	1.04	13.94	eV	(29)
melting point^b^	7316 (10765)	–196.0	492.5	°C	(10,30)

database	*n*	class ratio	reference
BBBP	1089 (2053)	161/928	(31)
Ames mutagenicity	3801 (6512)	1779/2022	(32)
hERG	2452 (6795)	1068/1385	(33)
ClinTox	707 (1478)	56/651	(28)

a The number of entries (*n*) is the number of molecules that lie within the applicability domain. The number in parentheses is the original number of molecules in the database.

b Melting point database is a combination of two other databases.

The most numerous database was the melting point database, which is a combination of two databases, and this database contains more than 7000 molecules that belong to the applicability domain. Two databases from the regression task databases contained fewer than 1000 molecules. The experimental properties had standard deviation values ranging from 1.04 to 2.18 except for the melting point, where the standard deviation was 94.09 °C. The classification databases contained binary activity classes, either active or inactive. Among the databases, two were highly imbalanced: the minority-to-majority ratios were approximately 1:12 in the case of the ClinTox database and 1:6 in the case of the BBBP database.

Based on the experimental data, a random forest algorithm implemented from the scikit-learn Python module with 500 estimators and maximum features parameters set to 0.3 and defined random seeds was used to model the experimental properties or activities. Ten times repeated 10-fold cross-validation was applied to assess the predictive performance of the models. The performance was evaluated by a set of metrics. In the case of regression, Pearson’s coefficient (*R*^2^) and root mean squared error (RMSE) were calculated. The classification performance metrics included the balanced accuracy and the area under the receiver operating characteristic curve (ROC AUC).

To fully assess the predictive performance of the QAPs, the achieved metrics were compared with the predictive performances of the baseline and the traditional chemoinformatics methods implemented in the RDKit Python module: Morgan fingerprints, atom pairs, topological torsions, RDKit fingerprints, and all molecular descriptors that are available in the RDKit.

In the case of regression tasks, the baseline models were models that, as a prediction, always returned the mean value of the training data set. In the case of classification, the baseline models always returned the majority class of the training data set.

Due to the high dimensionality of the data and the possible presence of noise, generated features that showed a standard deviation less than 0.05 were dismissed.

## Results and Discussion

The results indicate that in most cases QAPs provide enough information to yield predictions more accurately than does the baseline. Among the designed QAPs, spQAPs proved to be the most informative; thus, this type of representation was the most investigated. In some cases, enriching atom pairs with information derived from quantum chemistry appears to improve the accuracy compared to using traditional atom pairs.

**Figure 1:**
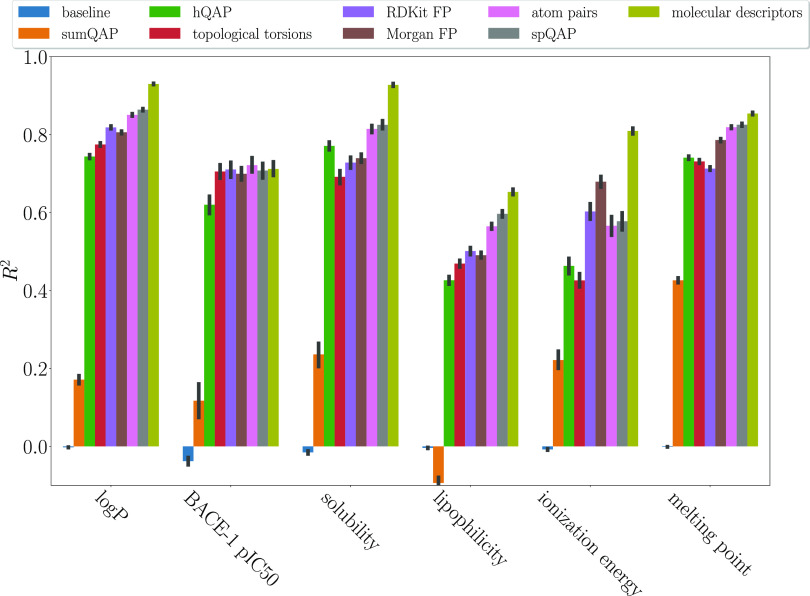
*R*^2^ of the regression task modeling. The black lines represent the 95% confidence intervals.

**Figure 2:**
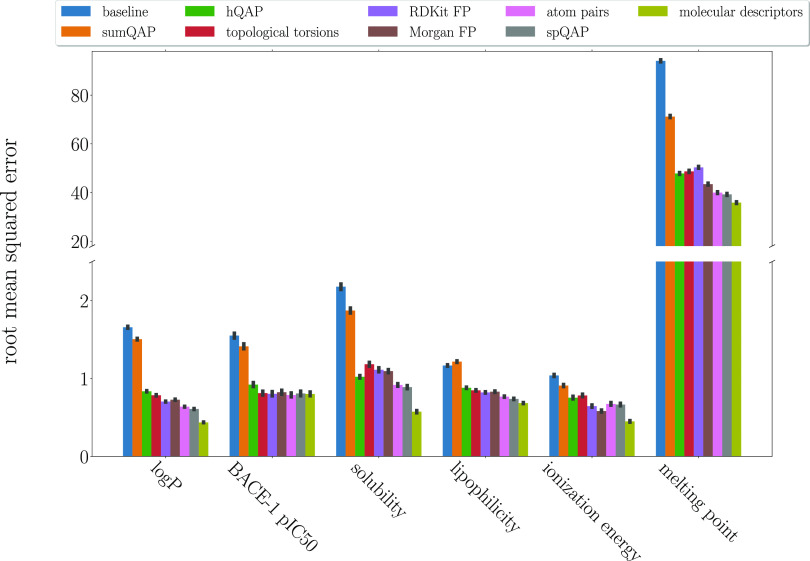
RMSE of the regression task modeling.

Considering regression tasks, based on Figures 1 and 2, which represent *R*^2^ and RMSE, respectively, the overall impression is that the best model performance is achieved with molecular descriptors, followed by spQAP and then unmodified atom pairs. The most informative molecular representations were atom pairs for predicting only the BACE-1 pIC50. Another exception was ionization energy, as Morgan fingerprints and RDKit fingerprints were better suited to create structure–property relationships.

**Figure 3:**
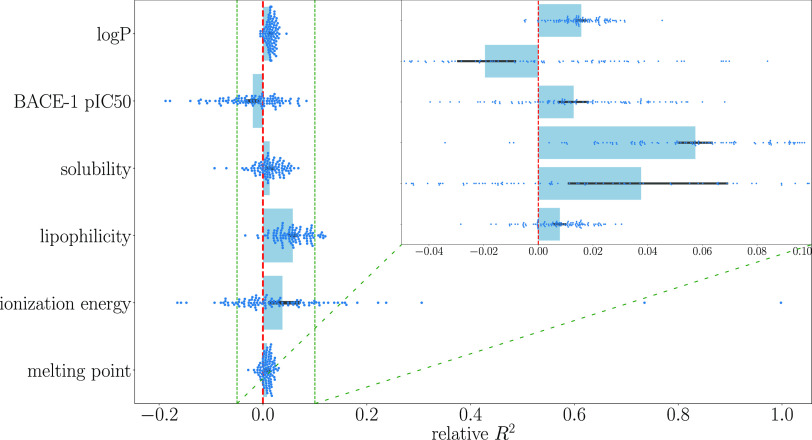
*R*^2^ of spQAP relative to the atom pairs descriptor. The greatest increase-approximately 6%—was observed in the lipophilicity database. The black lines represent 95% confidence intervals. Individual points are also shown as blue dots.

**Figure 4:**
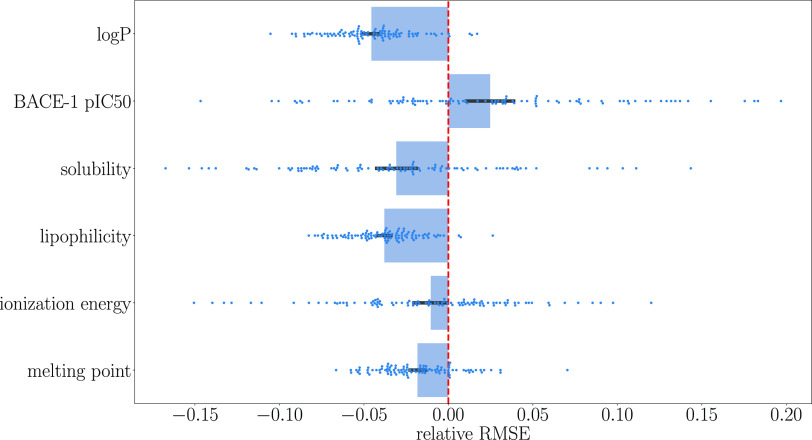
RMSE of spQAP relative to atom pairs descriptor. The average RMSE decreases most in the regression of log *P*.

To gain more insight into the differences in the metrics obtained with spQAP and atom pairs, the relative metrics were calculated as the ratio of the metric value obtained with spQAP to the value obtained with atom pairs minus 1. The ratio was calculated for each cross-validation fold and is visualized in Figures 3 and 4. The quantum modification of atom pairs caused an average *R*^2^ increase from 0.5 to 6%, and the RMSE decreased from approximately 0.5–5%, depending on the predicted property. In the case of predicting the BACE-1 pIC50, the average decrease in *R*^2^ was 2% and the RMSE increased by approximately 3%.

**Figure 5:**
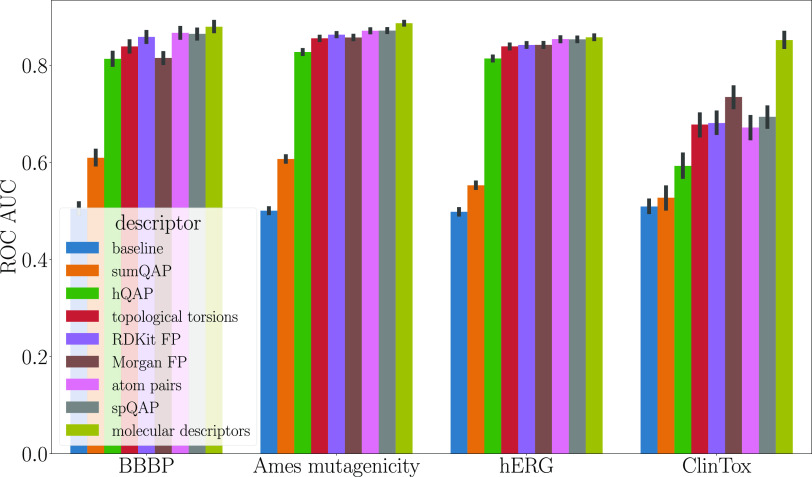
ROC AUC scores obtained for classification of the four databases.

Based on the ROC AUC (Figure 5), classification metrics obtained from the modelings resulted in trends similar to those in the regression tasks. That is, the best fits of the models are obtained when the structures are encoded as molecular descriptors and the second and third best results come from encoding structures such as spQAP and atom pairs, respectively. One exception is the classification of ClinTox database; the three best-performing representations are molecular descriptors, Morgan fingerprints, and spQAP.

**Figure 6:**
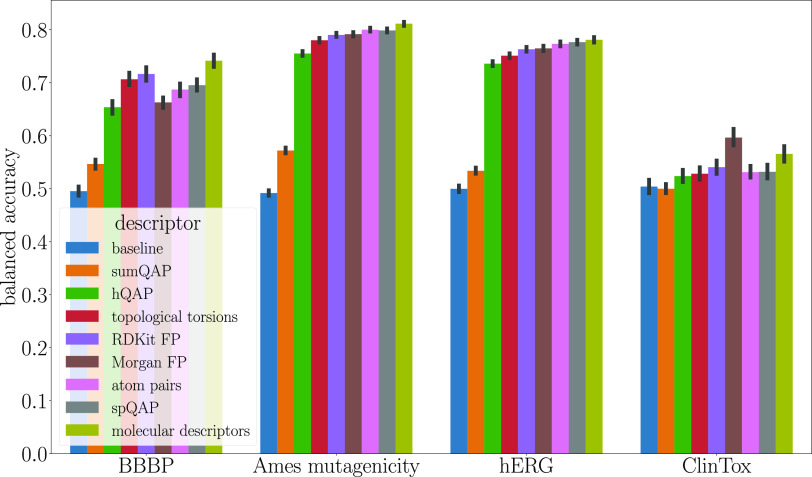
Balanced accuracy of the classification of four databases.

The results presented by the ROC curve are not fully consistent with the results of balanced accuracy (Figure 6); in the case of the BBBP data set, the three best-performing models are built from molecular descriptors, RDKit fingerprints, and topological torsions, and in the case of the ClinTox data set, the three best-performing representations are Morgan fingerprints, molecular descriptors, and RDKit fingerprints. This phenomenon may result from the imbalanced nature of the BBBP and ClinTox data sets. Although the ROC AUC is widely used in evaluating classifiers, there are reports that it may be misleading when the data set is imbalanced.^34^

To complement the results, a U.M.-W. test was applied to assess whether the results of the metrics varied between spQAP and the atom pairs. At the significance level of 0.05, the test indicated that the results are significantly different in the case of predicting the log *P*, the lipophilicity, and the melting point. This may indicate that the addition of quantum information to atom pairs is more effective in the regression tasks.

## Conclusions

In this research, novel molecular descriptors were introduced by modifying traditional atom pairs to contain quantum-chemical information. The greatest improvements in the validation metrics were obtained when the models performed regression tasks. In 5 out of the 6 databases, there was an improvement in the mean values of the metrics. The U Mann–Whithney test confirmed that in 3 cases the results obtained via cross-validation were significantly different. In contrast to quantum-chemical calculations of whole molecules and their application as molecular descriptors, the generation of quantum atom pairs is a rapid and easy process.

Despite boosting standard atom pairs with quantum information, the number of quantum atom pairs is limited, and further research should be conducted to overcome this problem. A possible solution is the creation of a larger database of quantum properties that may contain molecules built of more chemical elements than the QM9-extended-plus database. Other modifications of the quantum atom pairs may be achieved by calculating more than 11 quantum properties present in the QM9-extended-plus database.

## Data Availability

The QM9-extended-plus database is available at Zenodo.^25^ The code in the form of Jupyter Notebooks and Python scripts is available at GitHub.^35^ Data sets limited to the applicability domain are attached to this article.

## Supporting Information Available

Data sets curated to the applicability domain that were used (ZIP)
